# Short DNA/RNA heteroduplex oligonucleotide interacting proteins are key regulators of target gene silencing

**DOI:** 10.1093/nar/gkab258

**Published:** 2021-04-30

**Authors:** Ken Asada, Fumika Sakaue, Tetsuya Nagata, Ji-chun Zhang, Kie Yoshida-Tanaka, Aya Abe, Makiko Nawa, Kazutaka Nishina, Takanori Yokota

**Affiliations:** Department of Neurology and Neurological Sciences, Graduate School of Medical and Dental Sciences, Tokyo Medical and Dental University, 1-5-45 Yushima, Bunkyo-ku, Tokyo 113-8519, Japan; Center for Brain Integration Research, Tokyo Medical and Dental University, 1-5-45 Yushima, Bunkyo-ku, Tokyo 113-8519, Japan; Department of Neurology and Neurological Sciences, Graduate School of Medical and Dental Sciences, Tokyo Medical and Dental University, 1-5-45 Yushima, Bunkyo-ku, Tokyo 113-8519, Japan; Center for Brain Integration Research, Tokyo Medical and Dental University, 1-5-45 Yushima, Bunkyo-ku, Tokyo 113-8519, Japan; Department of Neurology and Neurological Sciences, Graduate School of Medical and Dental Sciences, Tokyo Medical and Dental University, 1-5-45 Yushima, Bunkyo-ku, Tokyo 113-8519, Japan; Center for Brain Integration Research, Tokyo Medical and Dental University, 1-5-45 Yushima, Bunkyo-ku, Tokyo 113-8519, Japan; Department of Neurology and Neurological Sciences, Graduate School of Medical and Dental Sciences, Tokyo Medical and Dental University, 1-5-45 Yushima, Bunkyo-ku, Tokyo 113-8519, Japan; Center for Brain Integration Research, Tokyo Medical and Dental University, 1-5-45 Yushima, Bunkyo-ku, Tokyo 113-8519, Japan; Department of Neurology and Neurological Sciences, Graduate School of Medical and Dental Sciences, Tokyo Medical and Dental University, 1-5-45 Yushima, Bunkyo-ku, Tokyo 113-8519, Japan; Center for Brain Integration Research, Tokyo Medical and Dental University, 1-5-45 Yushima, Bunkyo-ku, Tokyo 113-8519, Japan; Department of Neurology and Neurological Sciences, Graduate School of Medical and Dental Sciences, Tokyo Medical and Dental University, 1-5-45 Yushima, Bunkyo-ku, Tokyo 113-8519, Japan; Center for Brain Integration Research, Tokyo Medical and Dental University, 1-5-45 Yushima, Bunkyo-ku, Tokyo 113-8519, Japan; Laboratory of Cytometry and Proteome Research, Nanken-Kyoten and Research Core Center, Tokyo Medical and Dental University, 1-5-45 Yushima, Bunkyo-ku, Tokyo 113-8510, Japan; Department of Neurology and Neurological Sciences, Graduate School of Medical and Dental Sciences, Tokyo Medical and Dental University, 1-5-45 Yushima, Bunkyo-ku, Tokyo 113-8519, Japan; Center for Brain Integration Research, Tokyo Medical and Dental University, 1-5-45 Yushima, Bunkyo-ku, Tokyo 113-8519, Japan; Department of Neurology and Neurological Sciences, Graduate School of Medical and Dental Sciences, Tokyo Medical and Dental University, 1-5-45 Yushima, Bunkyo-ku, Tokyo 113-8519, Japan; Center for Brain Integration Research, Tokyo Medical and Dental University, 1-5-45 Yushima, Bunkyo-ku, Tokyo 113-8519, Japan

## Abstract

Antisense oligonucleotide (ASO)-based therapy is one of the next-generation therapy, especially targeting neurological disorders. Many cases of ASO-dependent gene expression suppression have been reported. Recently, we developed a tocopherol conjugated DNA/RNA heteroduplex oligonucleotide (Toc-HDO) as a new type of drug. Toc-HDO is more potent, stable, and efficiently taken up by the target tissues compared to the parental ASO. However, the detailed mechanisms of Toc-HDO, including its binding proteins, are unknown. Here, we developed native gel shift assays with fluorescence-labeled nucleic acids samples extracted from mice livers. These assays revealed two Toc-HDO binding proteins, annexin A5 (ANXA5) and carbonic anhydrase 8 (CA8). Later, we identified two more proteins, apurinic/apyrimidinic endodeoxyribonuclease 1 (APEX1) and flap structure-specific endonuclease 1 (FEN1) by data mining. shRNA knockdown studies demonstrated that all four proteins regulated Toc-HDO activity in Hepa1–6, mouse hepatocellular cells. *In vitro* binding assays and fluorescence polarization assays with purified recombinant proteins characterized the identified proteins and pull-down assays with cell lysates demonstrated the protein binding to the Toc-HDO and ASO in a biological environment. Taken together, our findings provide a brand new molecular biological insight as well as future directions for HDO-based disease therapy.

## INTRODUCTION

Antisense oligonucleotide (ASO)-based therapies have emerged for over the past decades (1). To data, several ASO drugs have been approved for the treatment of intractable diseases such as cytomegalovirus (CMV) retinitis, neovascular age-related macular degeneration (AMD), homozygous familial hypercholesterolemia, spinal muscular atrophy, and others (2,3). Another steric type ASO targeting exon skipping in Duchenne muscular dystrophy is in clinical trial (4). Chemical modifications of ASOs, including phosphorothioate (PS), 2′-*O*-methoxyethyl (MOE), 2′-*O*-methyl, 2′-fluoro, morpholino, peptide nucleic acid (PNA) and locked nucleic acid (LNA), have been investigated and are known to impact a pharmacokinetic advantage *in vivo* (5). We successfully achieved the target mRNA suppression with α-tocopherol (Toc) conjugated siRNA (6–9), and ASO characterization for the efficient oligonucleotides delivery (10). In 2015, we demonstrated a Toc conjugated DNA/RNA heteroduplex oligonucleotide (Toc-HDO) is more potent than the parent single-stranded gapmer ASO, achieving up to 95% reduction of the target liver *ApoB* mRNA *in vivo* (11), OAT3 mRNA expression regulation in the endothelial cells at the blood brain barrier in 2018 (12), and miR-122 expression suppression achieving 12-fold more effective in the liver in 2019 (13). Taken together, these studies indicate Toc-HDO therapeutic potential to target many classes of diseases.

It is well established that antisense drugs are RNase H-dependent and cleave their target transcripts in the nucleus associating with intracellular proteins in each step (incorporation, cellular trafficking, and cleavage of the target mRNA etc). Recently, RNase H1-dependent ASO gene targeting has been demonstrated in both the cytoplasm and nucleus (14). Crooke and other groups have identified ASO-binding proteins and characterized their functions and intercellular localizations (15–21). However, to date, no studies have shown that proteins can bind and regulate Toc-HDO activities. The identification of Toc-HDO-specific-binding proteins is of great importance to accelerate the Toc-HDO-based therapy. In addition, we believe that different intracellular trafficking mechanism of HDO from ASO could be responsible for its enhanced silencing activity. Thus, in order to identify Toc-HDO-binding proteins, ideally *in vivo*, we intravenously injected mice with Alexa 647-labeled Toc-HDO targeting *MALAT1* to identify potential binding proteins. Then, we performed several *in vitro* assays to investigate the binding profiles of identified proteins.

Here, we developed a comprehensive approach and identified Toc-HDO interacting proteins; annexin A5 (ANXA5), carbonic anhydrase 8 (CA8), apurinic/apyrimidinic endodeoxyribonuclease 1 (APEX1), and flap structure-specific endonuclease 1 (FEN1), that regulated the Toc-HDO function and may form a complex to regulate Toc-HDO-dependent gene silencing in multiple steps.

## MATERIALS AND METHODS

### General information

The chemical structure of Toc-HDO has been previously reported (11). ASO sequences targeting mouse *Malat1*, *DMPK*, *SRB1* and *ApoB* have also been reported (11,22–27). The LNA-DNA gapmers used in this study are as below (5′→3′ orientation):

*Malat1*:

ASO: 5(L)^∧^t(L)^∧^a(L)^∧^g^∧^t^∧^t^∧^c^∧^a^∧^c^∧^t^∧^g^∧^a^∧^a^∧^t(L)^∧^g(L) ^∧^5(L)

biotin-ASO: biotin-TEG-5(L)^∧^t(L)^∧^a(L)^∧^g^∧^t^∧^t^∧^c^∧^a^∧^c ^∧^t^∧^g^∧^a^∧^a^∧^t(L)^∧^g(L)^∧^5(L)

ASOPO: 5(L)t(L)a(L)gttcactgaat(L)g(L)5(L)

Toc- cRNA: Toc-g(M)^∧^c(M)^∧^a(M)^∧^uucagugaac^∧^u(M) ^∧^a(M)^∧^g(M)

Toc-PS cRNA: Toc-g(M)^∧^c(M)^∧^a(M)^∧^u^∧^u^∧^c^∧^a^∧^g^∧^u^∧^g^∧^a^∧^a^∧^c^∧^u(M)^∧^a(M)^∧^g(M)

Toc-PO cRNA: Toc-g(M)c(M)a(M)uucagugaacu(M)a(M)g(M)

*DMPK*:

ASO: a(L)^∧^5(L)^∧^a(L)^∧^a^∧^t^∧^a^∧^a^∧^a^∧^t^∧^a^∧^c^∧^c^∧^g^∧^a(L) ^∧^g(L)^∧^g(L)

Toc-cRNA: Toc-c(M)^∧^c(M)^∧^t(M)^∧^cgguauuuau^∧^u(M) ^∧^g(M)^∧^u(M)

*SRB1*:

ASO: T(L)^∧^5(L)^∧^a^∧^g^∧^t^∧^c^∧^a^∧^t^∧^g^∧^a^∧^c^∧^t^∧^T(L)^∧^5(L)

Toc-cRNA: Toc-g(M)^∧^a(M)^∧^agucaugacu^∧^g(M)^∧^a(M)

*ApoB*:

ASO: G(L)^∧^5(L)^∧^a^∧^u^∧^u^∧^g^∧^g^∧^u^∧^a^∧^u^∧^T(L)^∧^5(L)^∧^A(L)

Toc-cRNA: Toc-u(M)^∧^g(M)^∧^a(M)^∧^auaccaau^∧^g(M) ^∧^c(M)

where, 5 indicates 5-methyl C, (L) indicates LNA, ^∧^ indicates phosphorothioate bond, Toc indicates tocopherol, (M) indicates 2′ *O*-methyl, and TEG indicates triethylene glycol.

Antibodies against ACTIN, ANXA2, ANXA5, APEX1, CA8, FEN1, RNase H1, and TPM3 were obtained from Abcam (APEX1 #ab137708 and FEN1 #ab153825 for immunoblots), GeneTex (APEX1 #GTX110558 for immunoprecipitation), ProteinTech (ANXA2 #11256–1-AP and CA8 #12391-1-AP for immunoblots and immunoprecipitation, ANXA5 #11060-1-AP and RNase H1 #15606-1-AP for immunoblots, and TPM3 #10737–1-AP for immunoprecipitation), Cell Signaling Technology (normal Rabbit IgG #3900 (DAE1) for immunoprecipitaion), and WAKO (ACTIN #017-24573 for immunoblot). DNase I (#043-31261) was purchased from WAKO and recombinant RNase H was procured from Invitrogen (#18021-014).

### Animal experiments

The experiments adhered to the ethical and safety guidelines for animal experiments of Tokyo Medical and Dental University (#0140144A). All animal experiments were performed as previously described with minor modifications (11,28). C57BL/6J male mice aged 4–5 weeks were purchased from Oriental Yeast, Japan and kept on a 12 h light/dark cycle in a pathogen-free animal facility with free access to food and water. The Alexa 647-labeled (5′ end labeling) 16-mer ASO, and Toc-cRNA were purchased from GeneDesign, Inc. For the drug preparation, all oligonucleotides were formulated in PBS. A total of 50 mg of ASO or Toc-HDO, prepared as the mixture of 5 mg of Alexa 647-labeled ASO plus 45 mg of non-labeled ASO or of 5 mg of Alexa 647-labeled Toc-HDO plus 45 mg of non-labeled Toc-HDO, was administered per kg mouse. PBS, Alexa 647-labeled ASO, or labeled Toc-HDO was administered to the mice No. 1 - 4 (PBS), No. 13 – 16 (ASO), or No. 17–20 (Toc-HDO), respectively, via tail vein injection.

### Sucrose density gradient fractionation

100 mg of liver tissue were lysed in 2.5 ml of lysis buffer A (10 mM KCl, 10 mM Tris–HCl pH 8.0, 2 mM MgCl_2_ and 0.05% NP-40) with a protease inhibitor cocktail (Roche #04693116001). Following incubation on ice for 20 min, tissues were lysed using a tight-fitting douncer with 40 strokes, followed by centrifugation at 13 000 rpm spin at 4°C for 10 min to remove cell debris. A sucrose gradient was created by adding 2 ml of 36, 29, 22 and 15% sucrose to Beckman ultracentrifuge tubes and then 2 ml of the supernatant sample were added to the sucrose gradient to make up the final volume to 10 ml. A discontinuous gradient of 15–36% was formed and the tubes were centrifuged at 40 000 rpm for 5 h at 4°C (Beckman SW 41 Ti swing-bucket rotor). From the top of each gradient, 800 μl gradient fractions were collected to yield a total of 12 fractions. The Alexa fluorescence intensity of the fractions was measured using TECAN infinite M1000 Pro (TECAN).

### Identification of Toc-HDO binding proteins

Native polyacrylamide gel electrophoresis (PAGE) was performed with the sucrose density gradient fractions, followed by the visualization with EtBr for nucleic acids and with Coomassie Brilliant Blue (CBB) for proteins. The visualized bands were excised using a razor blade and analyzed by mass spectrometry at the Cellular and Proteome Research Laboratory at Tokyo Medical and Dental University.

### Native gel shift binding assay

Four volumes of lysis buffer A without NP-40 were added to liver tissue samples to obtain a liver extract. Ammonium sulfate precipitation was performed by saturating the extract with 30% salt. Following rotation for 1 h at 4°C, the supernatant fraction was resolved by centrifugation at 20 000 × *g* at 4°C for 15 min. The supernatant was dialyzed against a sufficient volume of buffer A for 2 h, followed by incubation with the Toc-HDO, ASO, or Toc-cRNA substrate for 20 min at 37°C. The incubated samples were resolved on native PAGE and the shift was visualized by EtBr staining.

### Preparation of shRNA, stable cell lines, and expression plasmid vector construction

The shRNA DNA oligos were cloned into the pBAsi-mU6 Pur vector (TaKaRa #3225). The non-targeting shRNA (shCtrl) and luciferase shRNA (shLuc) have been previously described (29–31). The shRNA sequences for each gene were chosen from validated sequence of Mission shRNA (Sigma-Aldrich) sequences. Mouse hepatocellular carcinoma Hepa1–6 cells were cultured in DMEM supplemented with 10% FBS. shRNA-mediated stable knockdown of cells was performed with Lipofectamine 2000 and selected using puromycin (1 μg/ml).

### Quantitative real-time PCR assay

Total RNA was extracted from cultured cells using QIAzol (Qiagen #79306) and then treated with DNase I. The purified RNA was reverse transcribed using Transcriptor Universal cDNA Master (Roche #05893151001) to synthesize cDNA. Expression levels were analyzed using the primers and probes for mouse *Malat1*, *DMPK*, *ApoB* and *Actin* (Applied Biosystems; Mm01227912_s1, Mm00446261_m1, Mm01601216_m1 and Mm00607939_s1, respectively) with a Light Cycler 480 Real-time PCR Instrument (Roche Diagnosis). To evaluate Toc-HDO and ASO cellular uptake efficiency by stable cell lines, quantitative real-time PCR assays were performed using a TaqMan MicroRNA Reverse Transcription Kit (Applied Biosystems #4366597) as previously described (11).

### Preparation of recombinant proteins and purification

Proteins ANXA5, CA8 and APEX1 were expressed in BL21 (DE3) cells using the pET 28a vector, while FEN1 was expressed using the pGEX6T1 vector. Cultures were grown at 37°C to an OD_600_ of 0.4–0.5. Induction of protein expression was performed with 1 mM IPTG for 8 h at 32°C. Cells were collected and washed with PBS. Harvested cells were lysed and purified as previously described with minor modifications (30). For FEN1 purification, the lysates were incubated with GST beads (GE Healthcare #17-0756-01) for 5 h and washed with PBS seven times. After the final wash, the GST was cleaved using the PreScission protease (GE Healthcare #27084301) for overnight at 4°C. All sequences used for subcloning are detailed in Supplementary Table S1.

### *In vitro* binding assays

Recombinant proteins were incubated with 1 μM of Toc-HDO, HDO, ASO, or Toc-cRNA for 20 min at 37°C in N buffer (100 mM NaCl, 20 mM Tris-acetate pH 8.0, 0.1 mM EDTA and 0.01% Triton X), M buffer (40 mM KCl, 20 mM Tris–HCl pH 7.8, 8 mM MgCl_2_ and 1 mM DTT), Buffer 1 (10 mM KCl, 10 mM Tris–HCl pH 8.0 and 2 mM MgCl_2_), or Buffer 17 (100 mM K-acetate, 15 mM HEPES pH 7.4 and 2.5 mM EDTA), then resolved on 13% PAGE and visualized by EtBr staining.

### Fluorescence polarization assays

Fluorescently labeled Toc-Malat1, Malat1 and ASO were synthesized by GeneDesign.

Purified recombinant proteins and Alexa 647-labeled nucleic acids were incubated at 37°C for 30 min with a total volume of 50 μl. Complexes were excited with polarized light at 635 nm and emission light at 675 nm was measured as fluorescence polarization signals with 5 s per measurement data acquisition (MF20 single molecule fluorescence spectroscopy system, Olympus). *K*_d_ values were calculated with Prism 8 (GraphPad) software using non-linear regression for curve fit assuming one binding site.

### Pull-down assays

Affinity selection with biotinylated ASOs has been reported (18,19) and affinity selection of Toc-HDO was performed with minor modifications. 50 μl of avidin agarose beads (Thermo Fisher Scientific #20219) were pre-incubated with 40 μl of 1200 μM biotinylated Toc-HDO or biotinylated ASO with 500 μl of PBS–0.05% Tween 20 for 2 h at 4°C. Unbound biotinylated Toc-HDO and ASO were removed by the centrifugation at 3000 rpm. Then, Toc-biotin-HDO- or biotin-ASO-bound avidin beads were added to the 1.4–2 mg of total cell lysates lysed by RIPA buffer. Samples were incubated over night at 4°C. The samples were washed with 1 ml PBS–0.01% Tween 20 five times. WB sample buffer was added to elute Toc-biotin–HDO or biotin–ASO binding proteins from the beads.

### Statistical analysis

Data are presented as mean ± SD. ANOVA/Dunnett's test was performed in R version 3.5.1 (Multcomp 1.4–13). * represents *P* < 0.05, ** represents *P* < 0.01 and *** represents *P* < 0.001.

## RESULTS

### The different distribution of Alexa-ASO and Alexa-Toc-HDO in sucrose density gradient fractionation

To identify the Toc-HDO binding proteins, Alexa-labeled ASO, or Alexa-labeled Toc-HDO were injected to the mice tail vein. Based on our preliminary experiments, cellular uptake efficiency was reached maximum concentrations in the mouse liver cells at 24 h post-administration in both ASO and Toc-HDO. Under this condition, we realized that there was a time gap between Toc-HDO trafficking and the target gene expression regulation. Thus, we decided to sacrifice the mice at the 3 h post-administration to identify multiple Toc-HDO interacting proteins.

**Figure 1.**
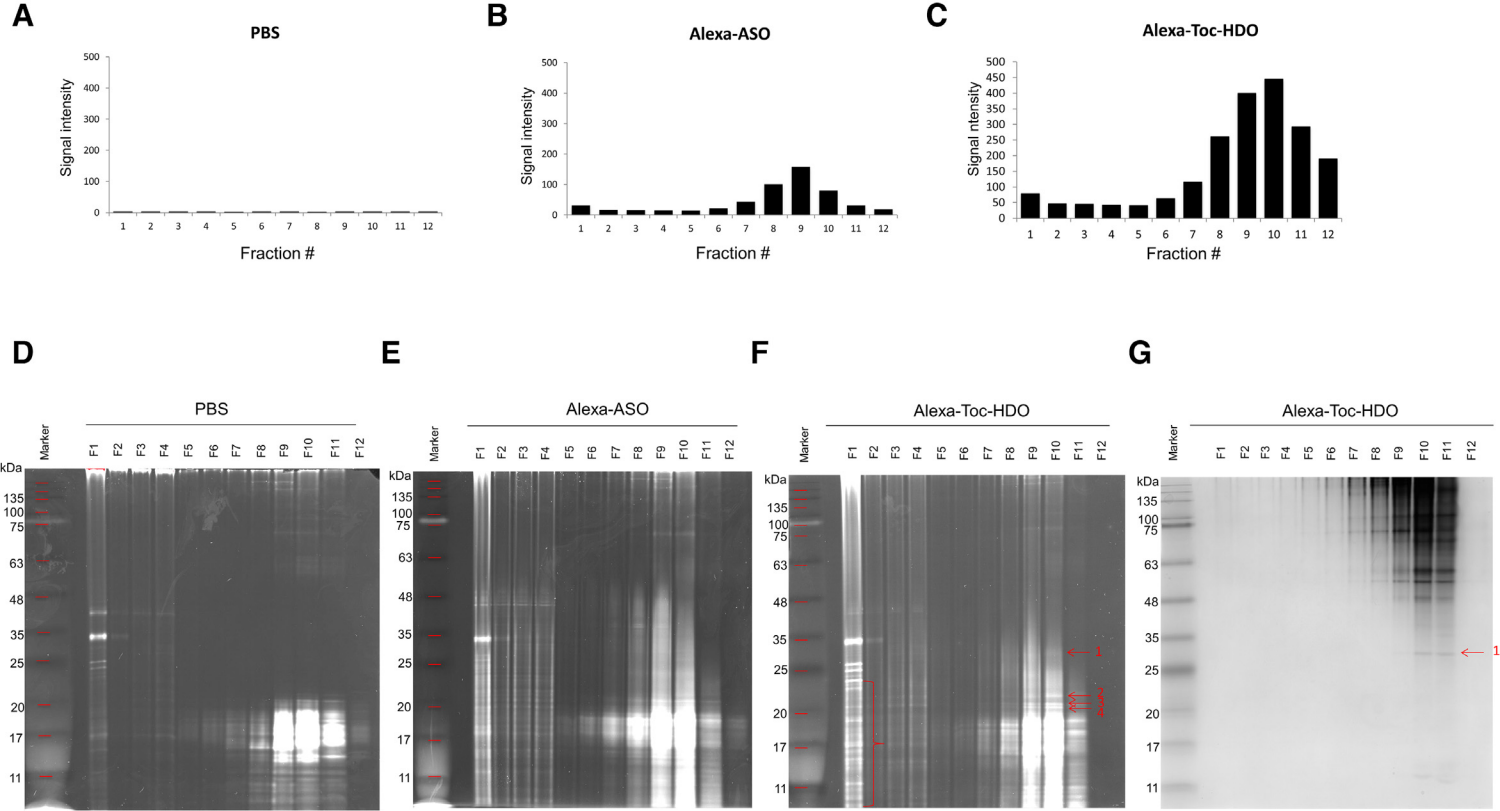
Fractionation-dependent differential distribution of Alexa-labeled ASO and Alexa-labeled Toc-HDO. (**A**–**C**) Fluorescence signal of fractionated liver extract from (A) PBS, (B) Alexa-labeled ASO, and (C) Alexa-labeled Toc-HDO intravenously administered mice. Data are representative of one of four experiments. (**D–F**) Fractionated (D) PBS, (E) Alexa-labeled ASO and (F) Alexa-labeled Toc-HDO treated liver samples were loaded onto non-denaturing polyacrylamide gels and nucleic acids were visualized by EtBr staining. (**G**) Proteins were visualized by CBB staining of the same gel of (F).

The mouse *Malat1*-targeting Toc-HDO used for the experiment was designed based on a previously reported (see Materials and Methods section). No signal was detected in the fractionated samples extracted from PBS-treated livers (Figure 1A). In contrast, Alexa 647-labeled ASO and Alexa 647-labeled Toc-HDO were mainly concentrated in fraction 9 (Figure 1B) and fraction 10 (Figure 1C) of the sample, respectively. These structure-dependent distributions suggest that Toc-HDO-specific binding proteins were localized in fraction 10. As expected, the signal obtained for Toc-HDO was stronger than that for ASO, as liver cells are known to show more efficient uptake (up to 5-fold) of Toc-HDO (11).

Next, to identify Toc-HDO-specific binding proteins, we aimed to visualize both the administrated Toc-HDO and fractionated proteins on the same gel by native PAGE. Theoretically, Toc-HDO and its binding proteins should co-migrate in the native gel. In agreement with the results presented in Figure 1C, Toc-HDO-specific nucleic acids were observed in fraction 10, compared with the control sample, as visualized by EtBr staining (Figure 1D for PBS, 1E for ASO and 1F for Toc-HDO). Resolved proteins were further visualized by CBB staining on the same gel and one clear band overlapping the EtBr-stained image was obtained (Figure 1G, arrow 1). Mass spectrometry analysis of the band indicated by arrow 1 revealed it the constituent as multiple proteins (Supplementary Table S2). Bands indicated by arrows 2, 3 and 4 did not appear on the CBB-stained gel, most likely because they were below the limit of detection.

### Identification of ANXA5 and CA8 as Toc-HDO binding proteins

**Figure 2.**
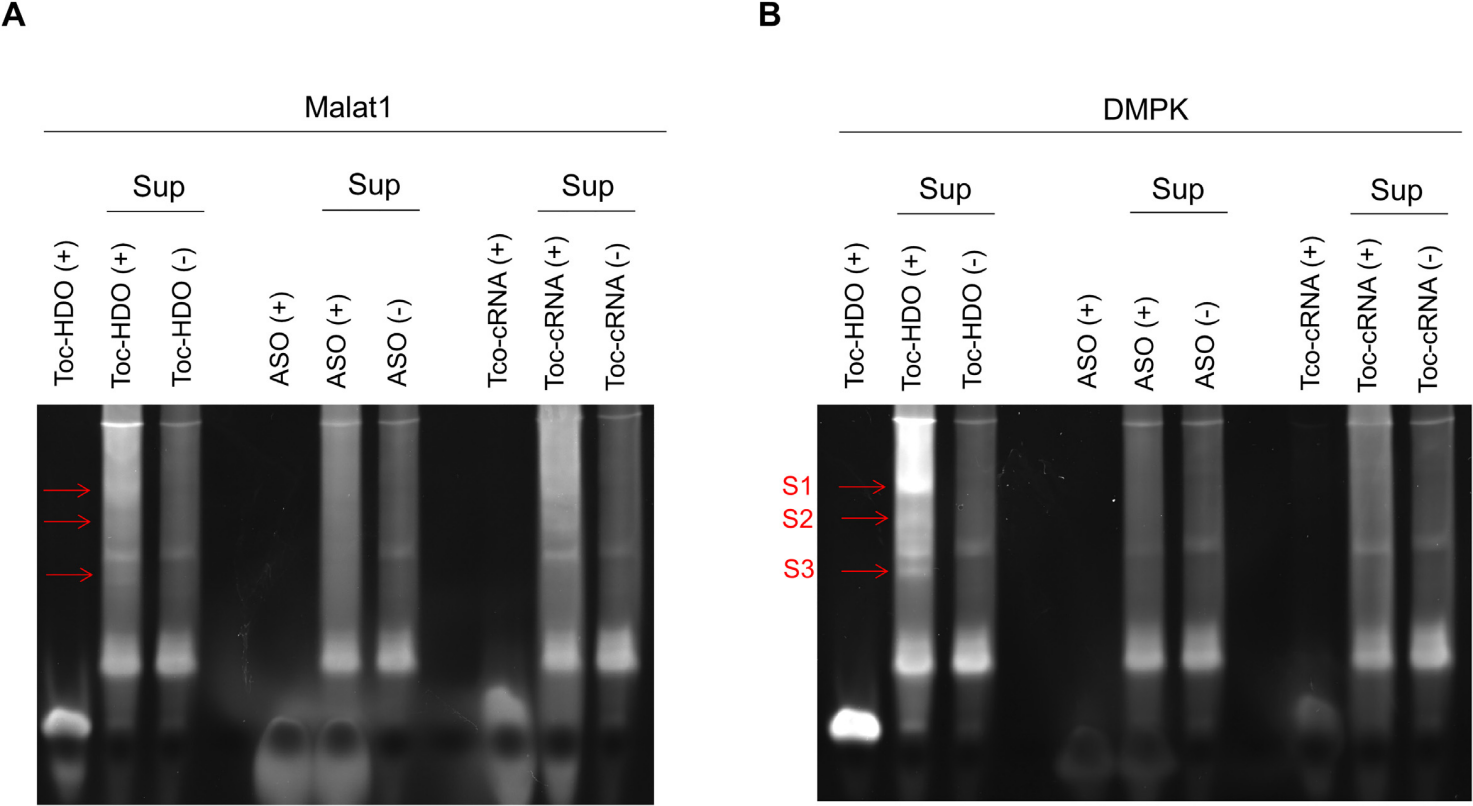
Native gel shift assays for the validation of proteins identified by mass spectrometry. (**A** and **B**) Toc-HDO binding assays with ammonium sulfate soluble fractions of (A) Toc-Malat1, or Malat1 ASO, or Toc conjugated complementary RNA (Toc-cRNA) and (B) Toc-DMPK, or DMPK ASO, or Toc-cRNA.

To determine whether the identified proteins are Toc-HDO interacting proteins, we conducted *in vitro* native gel shift assays (Figure 2). First, mice liver samples were extracted and fractionated by ammonium sulfate precipitation. Then, the soluble fractions were incubated with a Toc-HDO substrate targeting the *Malat1* gene, (hereafter referred to as Toc-Malat1) and three bands corresponding to their binding proteins were observed (Figure 2A, lane 2). Another sequence of Toc-HDO, targeting the *DMPK* gene (Toc-DMPK), gave a similar result compared with Toc-Malat1 (Figure 2B, arrows Supplementary Figures S1–S3). This indicated that Toc-HDO interacting proteins induced the observed shifts. Mass spectrometry analysis of arrows S1, S2 and S3 from Figure 2B is summarized in Supplementary Table S3. We identified two proteins by two independent methods with two Toc-HDO substrates; annexin A5 (ANXA5), a phospholipase A2 and protein kinase C inhibitory protein with calcium channel activity, and carbonic anhydrase 8 (CA8), a carbonic anhydrase family member lacking its activity, correspond to the arrow 1 band in Figure 1G and the S1 band in Figure 2B.

### Identification of two additional proteins that potentially bind with Toc-HDO

To identify more proteins that can bind Toc-HDO, we investigated previous work. More than 800 double-stranded DNA (dsDNA) or RNA/DNA hybrids interacting proteins were reported using the pull-down assays of biotinylated heteroduplex and streptavidin beads with B-cell extracts, followed by the LC–MS/MS analysis (32). Theoretically, identified over 800 proteins could bind to the Toc-HDO, however, it is not a realistic to examine all. Thus, we further investigated the annotated functions of all 803 proteins using NCBI gene (https://www.ncbi.nlm.nih.gov/gene) and then, we decided to focus on two proteins, apurinic/apyrimidinic endodeoxyribonuclease 1 (APEX1) and flap structure-specific endonuclease 1 (FEN1) because (i) both APEX1 and FEN1 interact with nucleic acids directly as a substrate (33–35), (ii) APEX1 and FEN1 are the only two proteins that are annotated as RNase H activity with previous studies (33,36–37) and (iii) partial sequence of FEN1 matches to the RNase H1 sequence (Supplementary Figure S1A–C) (38). Thus, we hypothesized that APEX1 and FEN1 could bind and regulate the Toc-HDO functions.

### Characterization of shRNA knockdown subcell lines

**Figure 3.**
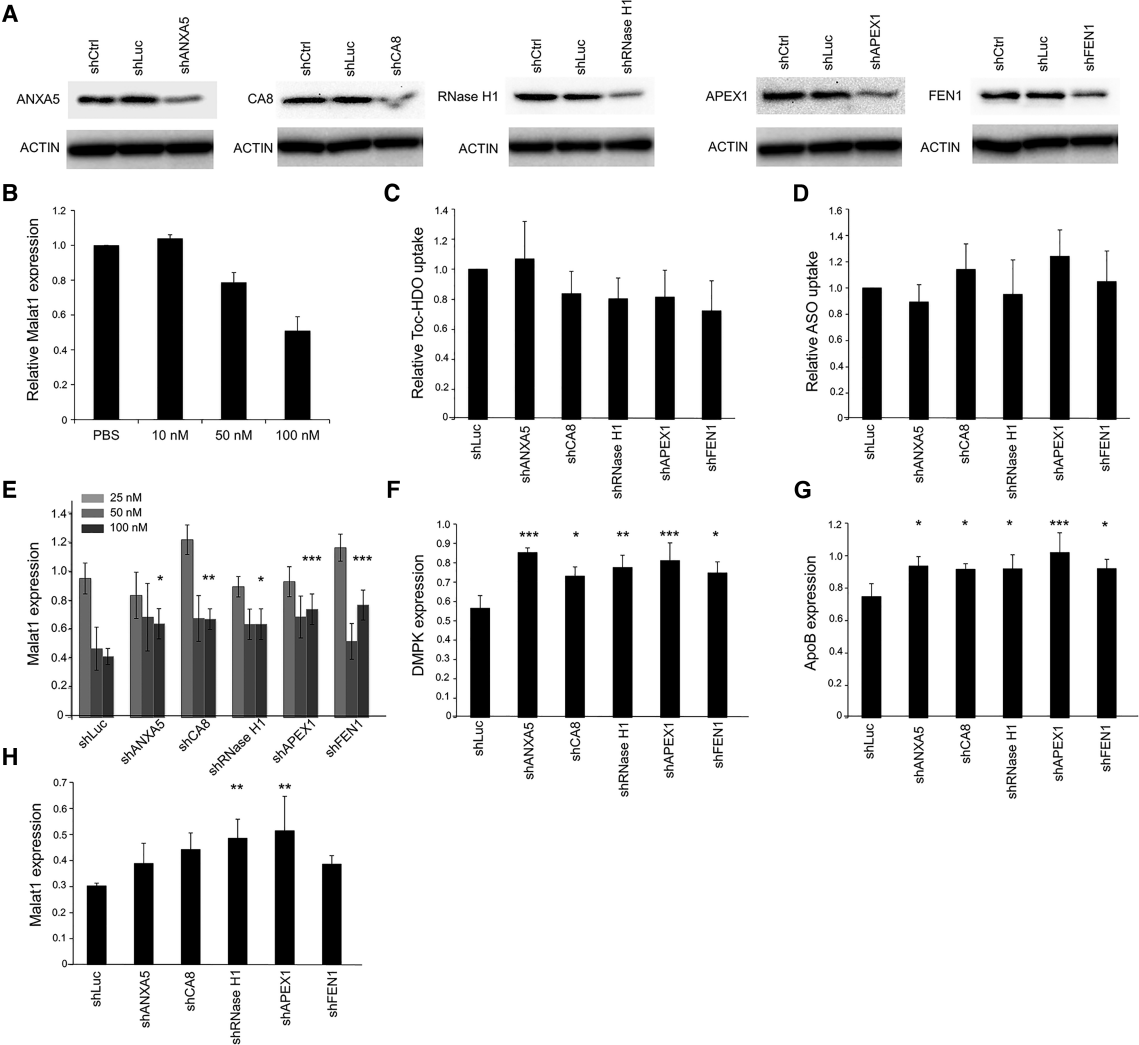
Toc-HDO-dependent gene silencing of the identified proteins. (**A**) Hepa1–6 cells were used to generate subcell lines stably expressing shRNA against no target (ctrl), *luciferase*, *ANXA5*, *CA8*, *RNase H1*, *APEX1* and *FEN1*. Immunoblots performed with the extracts from shRNA knocked down Hepa1–6 cells. (**B**) Toc-Malat1 dose-dependent gene expression regulation analyzed by RT-qPCR (*n* = 3). (**C** and **D**) Quantitative analysis of the substrates uptake of (C) Toc-Malat1 effect (*n* = 4), (D) ASO effect (*n* = 4). (**E**–**H**) Quantitative analysis of the (E) Toc-Malat1 effect with 25 nM (*n* = 3, light gray), 50 nM (*n* = 5, gray), and 100 nM (*n* = 4, dark gray), (F) 100 nM of Toc-DMPK effect (*n* = 4), (G) 100 nM of Toc-ApoB effect (*n* = 5) or (H) 500 nM of ASO effect against *Malat1* (*n* = 4).

To investigate whether the identified proteins regulate Toc-HDO-dependent gene expression silencing, subcell lines stably expressing shRNA against ANXA5 (shANXA5), CA8 (shCA8), RNase H1 (shRNase H1), APEX1 (shAPEX1) and FEN1 (shFEN1) were generated with shRNA controls (shCtrl and shLuc) (Figure 3A). Cells with a stable knockdown of the target genes were used to evaluate Toc-HDO-dependent target expression regulation. The dose-dependent target expression suppression is shown in Figure 3B. We mainly used 100 nM of Toc-Malat1 by gymnotic delivery to achieve nearly 50% knockdown of the *Malat1* expression to analyze the activation or suppression effect in the same percentage window for the future studies. To examine if cellular uptake was attenuated in the stable cells, gymnosis administered Toc-HDO and ASO (Figure 3C and D) were analyzed by RT-qPCR (11). Cellular uptake was normalized to the shLuc cells and we concluded that there were no significantly differences between the subcell lines.

### Toc-HDO activity in shRNA-mediated stable knockdown cells

We examined *Malat1* expression levels in shRNA knockdown cells treated by gymnosis. Although we observed a tendency of reduced activities of the Toc-HDOs in degrading the target *Malat1* RNA, there were no differences of the expression among cells in low- or middle-dose of Toc-Malat1 treatment (Figure 3E, light gray bar graph for 25 nM and gray bar graph for 50 nM). The effect of high-dose Toc-Malat1 treatment (100 nM) showed a *Malat1* expression upregulation in shANXA5-, shCA8-, shRNsae H1-, shAPEX1- and shFEN-knockdown cells than in cells treated with shLuc (Figure 3E, dark gray bar graph). These results indicate that ANXA5, CA8, RNase H1, APEX1 and FEN1 enhance Toc-HDO-dependent gene suppression in intact cells.

Next, to test whether those proteins can regulate another Toc-HDO activity, we aimed to knockdown the *DMPK* and *ApoB* transcripts, which are different sequences of Toc-Malat1. The expression levels of *DMPK* treated by Toc-DMPK and *ApoB* treated by Toc-ApoB were upregulated in all shRNA knockdown cells (Figure 3F and G). This suggests that the identified proteins regulate Toc-HDO activity in a sequence-independent manner.

We then evaluated the involvement of these proteins in the activity of ASO. ASO was administered to shRNA cells by gymnosis. Although we have seen a tendency of the *Malat1* upregulation in some cell lines, only shRNase H1 and shAPEX1 mitigated the gene suppression (Figure 3H). Our results indicated that ANXA5, CA8, RNase H1, APEX1 and FEN1 do regulate Toc-HDO activity and RNase H1 and APEX1 are the two proteins that regulate ASO activity in Hepa1–6 cells at the tested dose.

### Direct binding of ANXA5 and CA8 to Toc-HDO

**Figure 4.**
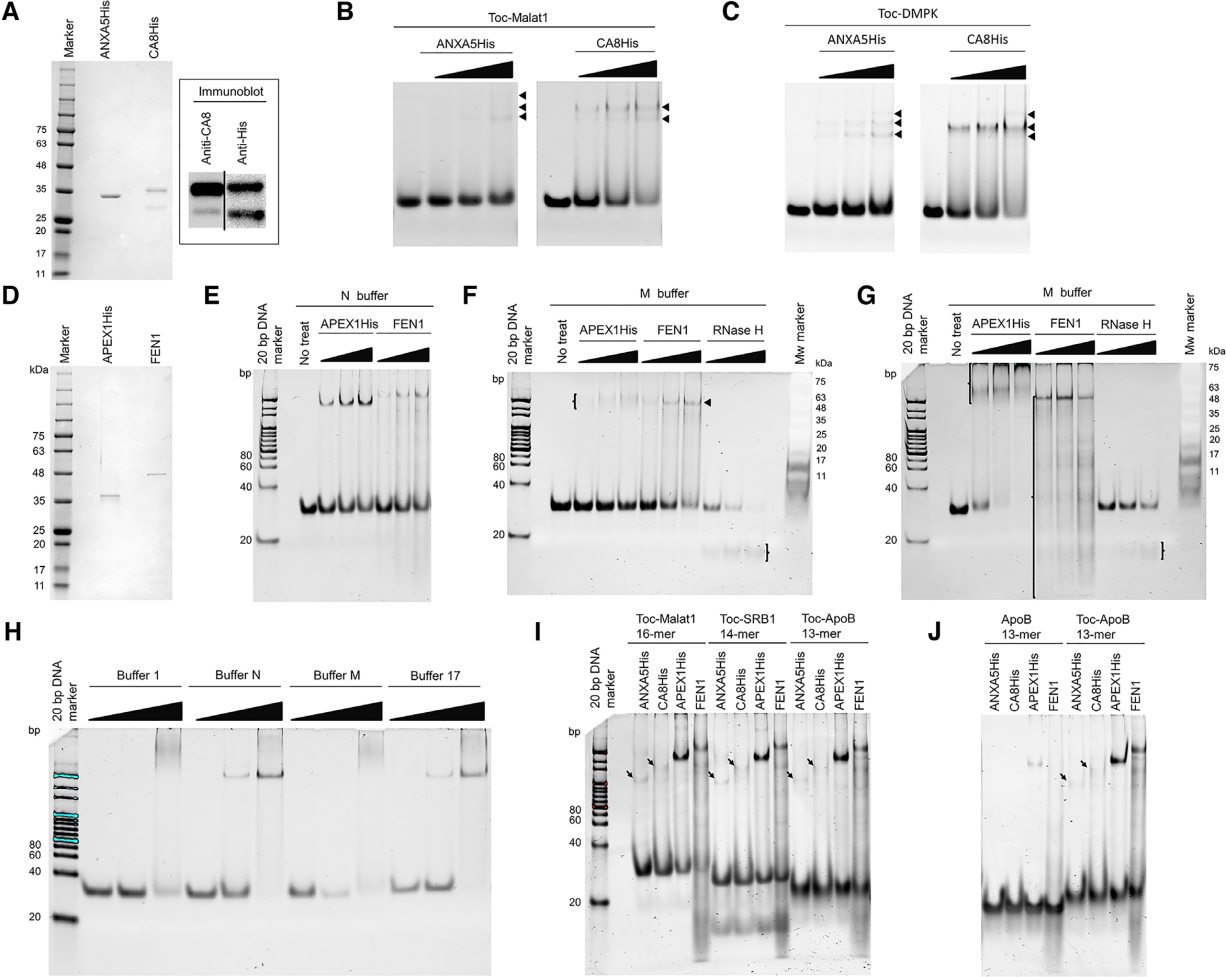
*In vitro* binding assays with purified recombinant proteins. (**A**) CBB stained polyacrylamide gel depicting purified recombinant ANXA5 and CA8 (left panel) and immunoblotting of recombinant CA8 proteins with either an anti-CA8 antibody or anti-His antibody (right panel). (**B** and **C**) Binding assays of recombinant ANXA5 (0, 250, 500 and 1000 nM) and CA8 (0, 500, 1000 and 2000 nM) with (B) Toc-Malat1 and (C) Toc-DMPK. (**D**) CBB stained polyacrylamide gel depicting purified recombinant APEX1 and FEN1. (**E** and **F**) *In vitro* binding assays of Toc-HDO substrates incubated with recombinant APEX1 and FEN1 in (E) N buffer (APEX1: 50, 100 and 150 nM; FEN1: 50, 100 and 150 nM), (F) M buffer with APEX1, FEN1 and RNase H (APEX1: 50, 100 and 150 nM; FEN1: 50, 100 and 150 nM; RNase H: 5, 10 and 20 mU). (**G**) Complete binding of Toc-HDO substrates by high dose of APEX1 and FEN1 in M buffer (APEX1: 100, 200 and 400 nM; FEN1: 100, 200 and 400 nM; RNase H: 1, 2.5, 5 mU). (**H**) Cation-dependent binding pattern of APEX1 (APEX1: 2.7, 27 and 270 nM). (**I**) Substrate length-dependent binding assays with ANXA5, CA8, APEX1 and FEN1 (1000, 1000, 150, 150 nM, respectively). (**J**) *In vitro* binding assays with or without Toc 13-mer substrates with ANXA5, CA8, APEX1 and FEN1 (1000, 1000, 150, 150 nM, respectively).

As the identified proteins regulated Toc-HDO activity in the cell, we decided to verify whether the proteins directly bind to the Toc-HDO or not by *in vitro* binding assays. We first generated ANXA5 and CA8 His-tag recombinant proteins (Figure 4A) because ANXA5 and CA8 were initially identified from the co-migration assays. Although recombinant CA8 appeared as two bands on the CBB-stained gel, both bands were detected by immunoblotting using anti-CA8 and anti-His antibodies, suggesting that both observed two bands were CA8. *In vitro* binding assays revealed the dose-dependent shifts for recombinant ANXA5 (Figure 4B, left) and CA8 (Figure 4B, right). As Toc-HDO sequence-independent target expression regulation was observed in Hepa1–6 cells, we further performed the assays with a different Toc-HDO substrate, Toc-DMPK (Figure 4C). *In vitro* binding assays demonstrated that the purified recombinant proteins bound Toc-Malat1 and Toc-DMPK similarly; further indicating that the identified proteins bind with a Toc-HDO, not with a sequence-dependent manner.

### Calcium-independent binding manner of ANXA5 and CA8

ANXA5 is known as a Ca^2+^-dependent phosphatidylserine-binding protein. CA8 is an inositol triphosphate (IP_3_) receptor-binding protein that inhibits IP_3_ binding to the IP_3_-binding receptor type 1 and modulates calcium signaling (39–41). These previous studies suggest that Ca^2+^ is associated with the cellular function of ANXA5 and CA8. Therefore, we performed *in vitro* binding assays to confirm whether Ca^2+^ enhances Toc-HDO-protein binding affinity. The presence of Ca^2+^ had not effect on the binding affinity of ANXA5 to Toc-Malat1 and Toc-DMPK (Supplementary Figure S2A). Furthermore, Ca^2+^ with EDTA, did not influence the binding affinity of ANXA5 and CA8 (Supplementary Figure S2B). Taken together, we conclude that Toc-HDO binding by ANXA5 and CA8 is Ca^2+^-independent.

### Toc-HDO binding and nuclease activity of APEX1, FEN1, and RNase H

Next, to examine whether APEX1 and FEN1 bind Toc-HDO directly, APEX1 and FEN1 were purified using His beads or GST beads, respectively, followed by GST cleavage using the PreScision protease (Figure 4D). In *in vitro* binding assays using a monocationic buffer (N buffer), the APEX1-Toc-HDO complex was observed as a single band and the FEN1–Toc-HDO complex was observed as a major band with smear bands in a dose-dependent manner (Figure 4E).

As we previously described, the reason why we decided to focus on APEX1 and FEN1 was that they are reported as function as RNase H activity. Thus, to test whether APEX1 or FEN1 have the nuclease activity to the Toc-HDO, we performed *in vitro* nuclease assays with MgCl_2_ buffer (M buffer), as RNase H is a dication-dependent nuclease. Under dicationic conditions, *Escherichia coli* RNase H showed clear Toc-HDO cleavage in M buffer (Figure 4F), suggesting that RNase H1 is at least, one of the Toc-HDO cleaving enzymes *in vitro*. On the other hand, APEX1 exhibited no cleaving activity and slightly weak binding affinity compared to when a monocationic buffer was used (Figure 4E: monocation; Figure 4F: dication). Furthermore, the binding complex of APEX1 was larger and smears. In contrast, FEN1 demonstrated similar binding affinity in N buffer and M buffer and there were smear bands detected especially in the highest dose of FEN1 (Figure 4F). Regarding the shifted band in FEN1 treated samples; Toc-HDO has a molecular weight (Mw) of 11 kDa, while the Mw of the recombinant FEN1 is 43 kDa. Thus, the observed shift may indicate that a single Toc-HDO molecule directly bound with FEN (11 kDa + 43 kDa = 54kDa). However, unlike RNase H1, we did not clearly detect any of specific degraded products of Toc-HDO. It is possible that the enzymatic activity of APEX1 and FEN1 might not be strong enough to cleave Toc-HDO compared with RNase H. In particular, RNase H activity in APEX1 is very weak compared to its apurinic/apyrimidinic (AP) endonuclease activity (35). Therefore, we conducted *in vitro* assays with higher concentrations of the proteins and a longer incubation time.

APEX1 and FEN1 showed in the complete binding of the Toc-HDO substrates (Figure 4G). At the highest dose of FEN1, the observed incorporated band became weaker than at the low or middle concentrations, indicating that Toc-HDO may be cleaved or degraded by FEN1 *in vitro* as Toc-HDO incubated without any recombinant protein remained stable; however, it is possible that the results obtained with a high dose of recombinant protein are physiologically irrelevant.

### APEX1 cation-dependent binding manner to the Toc-HDO

As the binding pattern of the APEX1 was cation-dependent, we aimed to clarify the effect of cations on APEX1 using four different buffers. The APEX1-Toc-HDO complex appeared to have a higher Mw when incubated with Mg^2+^ (Buffer 1 and Buffer M) than with monocationic Na^+^ or K^+^ (Buffer N and Buffer 17) (Figure 4H). This indicates that under the monocationic conditions, there is only a binding pattern detectable between APEX1 and Toc-HDO. On the other hand, under dicationic conditions, there seem to be several binding patterns exist, possibly, one APEX1 may bind multiple molecules of Toc-HDO or multiple APEX1 molecules may bind one molecule of Toc-HDO.

### Similar binding affinity of identified proteins to the 13 to 16-mer Toc-HDO

To determine whether the identified proteins have a length limitation for Toc-HDO recognition, we conducted *in vitro* binding assays with Toc-Malat1 (16-mer; 10-mer gap), Toc-SRB1 (14-mer; 10-mer gap), and Toc-ApoB (13-mer; 8-mer gap). Surprisingly, ANXA5, CA8, APEX1 and FEN1 bound 16-mer, 14-mer and 13-mer Toc-HDO with a similar binding affinity, indicating that there is no correlation to the length from 13- to 16-mer or 8- to 10-mer gap DNA (Figure 4I). We observed unhybridized SRB1 substrate at the bottom of the gel. The parental ASO of SRB1 has a palindromic sequence that caused a technical problem; this was introduced to generate a short hairpin structure as well as to hybridize a complementary RNA of ASO to generate a Toc-HDO structure.

### The functional role of Tocopherol to the 13-mer HDO

To test the importance of Toc for the HDO binding affinity, we performed *in vitro* binding assays with or without Toc to the 13-mer ApoB. The gel-resolved binding assays clearly showed that the absence of Toc significantly lowered the binding affinity of ANXA5, CA8, APEX1 and FEN1 (Figure 4J), suggesting that Toc enhances the binding affinity at least in the length of 13-mer.

### Characterization of binding affinity to the Toc-HDO, HDO and ASO using fluorescence polarization assays

**Figure 5.**
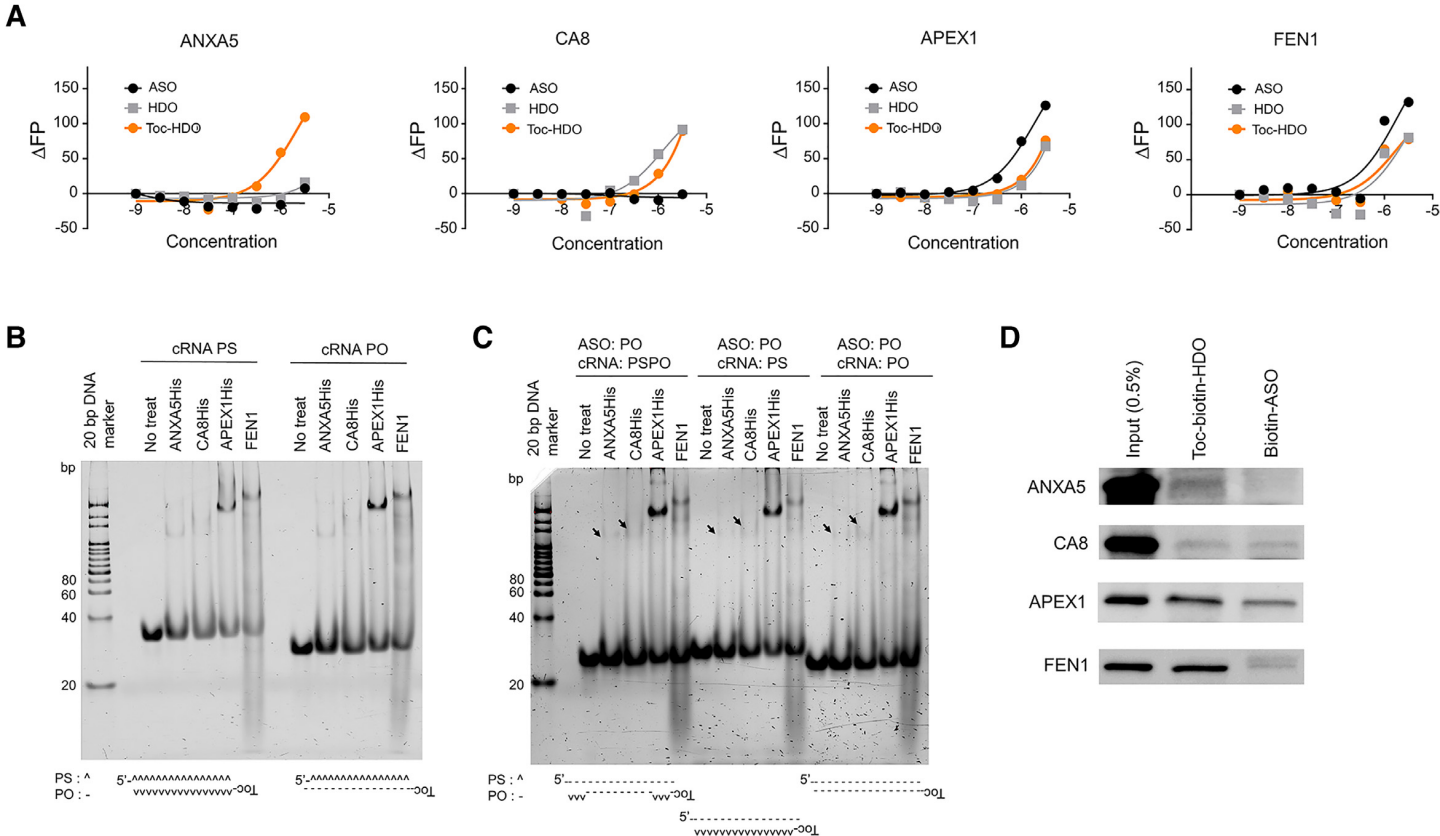
Characterization of identified proteins. (**A**) Fluorescence polarization assays of Toc-HDO substrates incubated with recombinant ANXA5, CA8, APEX1, and FEN1 proteins (10^−9^ to 10^−5.5^ M). (**B** and **C**) *In vitro* binding assays with ANXA5, CA8, APEX1, and FEN1 (1000, 1000, 150 and 150 nM, respectively) and Toc-HDO substrates with (B) PS ASO backbone and (C) PO ASO backbones. (**D**) Pull-down experiments using Toc-biotin–HDO and biotin–ASO of Malat1.

Although EtBr is widely applied to visualize nucleic acids in the laboratory, its detection limit is at the nanogram order and it is technically difficult to obtain high-resolution images, in particular, with single stranded nucleic acids. Therefore, we performed more precise fluorescence polarization (FP) assays to examine whether identified proteins bind not only Toc-HDO but also HDO or even ASO. Interestingly, the result of the FP assays showed that ANXA5 preferably bound with Toc-HDO and very low or no binding to HDO and ASO, indicating binding affinity is high to the Toc (Figure 5A), concordant with the result of Toc-ApoB and ApoB (Figure 4J). CA8 bound with Toc-HDO and HDO at the tested dose, indicating binding motif is a heteroduplex-dependent *in vitro*. APEX1 and FEN1 bound with Toc-HDO, HDO and ASO. FP assays demonstrated that the signal became detectable from submicromolar between CA8, APEX1 and FEN1 to the 16-mer Toc-Malat1 and Malat1. *In vitro* binding assays without Toc in 13-mer ApoB clearly reduced the binding affinity. Taken together, Toc plays two important roles in biology; (i) enhancing the binding affinity in 13-mer of the four identified proteins (ii) essential component for ANXA5 to bind Toc-HDO *in vitro*. Computed Kd values were summarized in Supplementary Table S4. Of note, we cannot exclude the possibility that *K*_d_ value might be inaccurate because we set the parameter as one molecule bind with one protein.

### Characterization of binding affinity with a different phosphate backbone of Toc-HDO

The profiling of ASO interacting protein shows that ASO function is highly affected by the length, charge content, and modifications (5,17,20,42). The Toc-HDO used for our experiments constituted an LNA–DNA–LNA gapmer with a full phosphorothioate (PS) backbone for ASO and phosphorothioate–phosphodiester (PS–PO) linkage for cRNA; thus, we characterized whether PS and PO Toc-HDO show different features to the binding proteins. First, we generated a full PS backbone of ASO hybridized with a full PS and full PO backbones of cRNA. All tested proteins were able to bind these Toc-HDOs and their binding affinities are similar between two cRNA backbones and slightly high in APEX1 with PO cRNA (Figure 5B). Second, we generated a full PO backbone of ASO hybridized with PS–PO, PS or PO stranded cRNA. ANXA5 and CA8 showed a weak binding compared with a PS-ASO backbone, this suggests PS backbone of ASO is preferable for ANXA5 and CA8 (Figure 5C). APEX1and FEN1 clearly bound these Toc-HDOs (Figure 5C).

### Pull-down assays to investigate the binding affinity to Toc-HDO and ASO in a biological environment

Although FP assays and *in vitro* biding assays showed a direct binding between proteins and Toc-HDO, HDO or ASO, it is uncertain whether these proteins bind with Toc-HDO and/or ASO in a biological environment. Therefore, we decided to perform pull-down experiments. ANXA5 and CA8 showed a weak affinity with Toc-biotin-HDO, which composed of 5′-biotin-conjugated ASO and 5′-Toc-conjugated cRNA (Figure 5D). Biotin-ASO barely bound with ANXA5 but showed a similar affinity to the CA8. Toc-HDO showed a stronger binding affinity to APEX1 and FEN1 compared with ANXA5 and CA8, which we have observed in Figure 4I, J, 5B, and C. Additionally, ASO binding affinity was affected in the presence of cellular proteins, at least to the CA8, because FP assays with recombinant CA8 demonstrated no binding to the ASO of Malat1.

### A new CA8-interacting protein identified by immunoprecipitation and mass spectrometry analyses

In order to elucidate the protein–protein interactions that may be associated with Toc-HDO trafficking, we performed immunoprecipitation (IP) analyses to pull-down ANXA5- and CA8-binding protein because ANXA5 and CA8 were identified from the mouse liver tissues at the time point of 3 h post Toc-HDO administration (Figure 1). Although we failed to precipitate ANXA5 by IP with two different antibodies (unpublished data), the CA8 antibody successfully precipitated CA8 proteins using RIPA buffer and nonionic detergent NP-40 buffer (Supplementary Figure S3A left; RIPA buffer, right; NP-40 buffer). As the precipitated protein yield was greater in NP-40 buffer than in RIPA buffer, we proceeded with NP-40 buffer for the identification of the CA8-binding proteins. The precipitated proteins were resolved on SDS-PAGE and visualized by silver staining. Two specific bands were observed in the IP samples. Sequential mass spectrometry analysis identified the novel CA8-binding protein as tropomyosin 3 (TPM3; Supplementary Figure S3B; arrow C2 and Supplementary Table S5), a member of the tropomyosin family of actin-binding proteins. To the best of our knowledge, this is the first evidence that CA8 binds to TPM3.

### Immunoprecipitation–western blotting analysis revealed protein–protein interactions between the identified proteins

Figure 5D shows CA8 bound with ASO in a biological environment, indicating that there are potential binding partners to enhance binding affinity to ASO and probably to the Toc-HDO. Thus, to elucidate the protein complexes related to the Toc-HDO and ASO, we performed immunoprecipitation-western (IP) experiments (Supplementary Figure S3C–E). We investigated the protein-protein interactions of the identified proteins, as well as with ANXA2 that is known to regulate ASO activity (15,43). The protein–protein interactions we revealed here could be associated with Toc-HDO gene silencing.

### Protein expression levels of identified proteins in Hepa1–6 cells

Next, to apply the knowledge of the *in vitro* assay results to the physiological aspect, we examined each protein expression levels in Hepa1–6. The total cell lysate was resolved on a gel with a known concentration of the purified recombinant proteins. Endogenous protein expression levels were 0.03–1 ng/μg, which required a reasonable exposure time (within 5 min) for expression detection by immunoblotting (Supplementary Figure S3F and G). In addition, albeit mass spectrometry analysis is a qualitative rather than quantitative, we obtained 35 peptides corresponding to the ANXA5 sequence and 5 peptides corresponding to CA8 from Figure 1G sample and 16 peptides corresponding to ANXA5 and two peptides corresponding to CA8 from Figure 2B sample (Supplementary Tables S2 and S3). The estimated protein expression levels of ANXA5 and CA8 were 0.38 and 0.03 ng/μg respectively, in mouse Hepa1–6 cells, which is perhaps associated with the mass analysis results for samples generated from mouse liver tissues. Taken together, the cellular expression levels of identified proteins are probably average.

## DISCUSSION

Our previous fluorescence images demonstrated that not only parenchymal cells but also non-parenchymal cells of liver cells uptake Toc-HDOs (11). Later, we quantitated the cellular uptake of single-stranded antimiR and double-stranded HDO-antimiR to the parenchymal cells and non-parenchymal cells (13), suggesting that without Toc, cells uptake both single-stranded and double-stranded nucleic acids with the similar efficiency.

The importance of nucleic acid therapy has surged over the past decades. Toc-HDOs are more potent and stable and are effectively delivered to their target tissues compared to the parental ASOs. Toc is important for a specific drug delivery and for enhancing binding affinity, and Toc-HDO could be recognized by not only the identified proteins but also other DNA sensor proteins (31), RNA sensor proteins, dsDNA and dsRNA binding proteins (44), or might be even Ago2 or RISC since DNA-guided DNA interference by Ago has been studied previously (45,46).

Heterogeneous protein–protein interactions have been observed between ANXA1 and ANXA2 as well as ANXA2 and ANXA5, as determined by fluorescence resonance energy transfer signals in transfected N1E-115 cells (47) and Ca^2+^-dependent ANXA2–ANXA5 interactions (48). To date, no nucleic acids binding ability has been reported for ANXA5 and CA8 proteins. Here, we identified multiple Toc-HDO-interacting proteins, ANXA5, CA8, APEX1 and FEN1, which regulated Toc-HDO activity targeting *Malat1*, *DMPK* and *ApoB*. We initially found that ANXA5 and CA8 interact Toc-HDO using a specific screen with co-migration assays. Subsequently, we identified ANXA5 as a Toc-HDO binding, and CA8 as a Toc-HDO and HDO binding protein using *in vitro* binding assays and FP assays. However, pull-down assays demonstrated that CA8 did bind with both Toc-HDO and ASO with a similar binding affinity, suggesting that there are potential binding partners in a biological environment to enhance the binding affinity to ASO. In our IP-mass spectrometry analysis using CA8 antibody, we identified a novel CA8 interacting protein TPM3. TPM3 was identified as an HSP90-AA1 interacting protein (49,50) that is one of the ASO binding proteins identified by pull-down assays (18) and later, it was reported that HSP90 protein enhances antisense activity of various PS-ASOs (19).

FP assays further demonstrated that APEX1 and FEN1 bound Toc-HDO, HDO and ASO. APEX1 and FEN1 were initially identified as double-stranded DNA (dsDNA) or RNA/DNA hybrids interacting proteins by using pull-down experiments, followed by the LC–MS/MS analysis (32) and here, we demonstrated that APEX1 and FEN1 bound with Toc-HDO.

Recently, we have developed the fluorescence resonance energy transfer (FRET) assays with live-cell time-lapse imaging to visualize how HDO transfected with RNAiMAX undergoes to the nucleus (51). These assays revealed that HDO had a different intracellular trafficking mechanism from ASO. Although we cannot extrapolate all the findings obtained by RNAiMAX transfected studies to the gymnosis administered Toc-HDO cellular trafficking, it is reasonable to assume that Toc-HDO could form a different complex than ASO. This indicates that further studies for a more comprehensive understanding of Toc-HDO binding proteins and Toc-HDO trafficking are required.

One limitation of our study is that there was a difference in binding affinities between the result of *in vitro* assays using recombinant proteins and the result of a biological environment, notably to the CA8 protein. Although the identified four proteins showed a Toc-HDO dependent gene regulation, further molecular mechanisms including biochemical assays and animal studies, particularly using knockout mice are needed.

Of note, like FEN1, the nuclease activity rises as much as 50-flod with the existence of binding partner (52), implying that *K*_d_ values *in vivo* might be different compared with *in vitro* result. Interestingly, ANXA5 binds with amyloid beta precursor protein (APP) and APP binds with APEX1 (53). ANXA5 also binds with heterogeneous nuclear ribonucleoprotein A1 (HNRNPA1) and HNRNPA1 binds with FEN1 (54,55). Furthermore, purified APEX1 and FEN1 directly bind each other (56), suggesting that proteins including identified proteins can form a protein complex.

Initially, PS was introduced as an RNA modification to increase stability. Recently, it is reported that PS structure is important in ASO-dependent gene targeting efficacy (57). The replacement of oxygen by sulfur at phosphorus sites raises a structural issue of the chirality. Actually, we do not exactly know about the configurations of the Toc-HDO used in the assays and the different configuration may affect the Toc-HDO activity by altering the binding affinity. To tackle this problem, computational simulation studies, X-ray crystallography or other suitable methodology could be required.

Previous studies demonstrated that APEX1 and FEN1 could function as an RNase H. RNase H1 has a consensus sequence that represented as DDED domain. None of the APEX1 sequence matched to the RNase H1 sequence and partial FEN1 sequence aligned only one of the conserved DDED domain of RNase H. This probably links to the result that APEX1 and FEN1 bound with but it is hardly seen a specific cleaved product of Toc-HDO. Dication-dependent *E. coli*. nuclease RNase H cleaved Toc-HDO in the presence of Mg^2+^ (M buffer), but failed to cleave in the presence of Na^+^ in our assays (N buffer, unpublished data), indicating that RNase H activity to the Toc-HDO is also Mg^2+^-dependent, concordant with the knowledge that is widely accepted. Despite *in vitro* assays demonstrated that RNase H cleaved the Toc-HDO, northern blot analysis of liver tissue samples treated with 13-mer DNA/31-mer cRNA overhang Toc-HDO for probe hybridization showed that major cleavage sites are different from the recombinant human RNase H1 did (11), indicating that other proteins do cleave Toc-HDO *in vivo*.

It is possible that small heteroduplex DNA/RNA and small single-stranded DNA can be generated endogenously, especially from the known DNA/RNA hybrids. DNA/RNA hybrids play important roles in both cytoplasm and nucleus and are observed as R-loop when cells are exposed to stalled DNA replication, DNA damage, and fork collisions. DNA/RNA hybrids are also observed as Okazaki-fragments during DNA replication, or during the reverse-transcription of retrotransposons. The length of previously reported DNA/RNA hybrids is predominantly ranged 100–1000 for dsDNA and <100 for single-stranded DNA (ssDNA). We recently demonstrated that cytosolic genomic DNA (cg721; 297 bp long) regulates its complementary RNA expression by ssDNA digesting enzyme TREX1-dependent manner (31). cg721 is detectable in physiological normal condition and serves as a natural antisense DNA.

However, it is not deeply investigated whether small DNA/RNA (13–20 bp) exists endogenously. Our protein profiling demonstrated that the identified proteins, at least APEX1 and FEN1 bind to the small DNA/RNA even they have a PO backbone, indicating that there might be a small DNA/RNA or small ssDNA biology in nature related to the gene expression regulation. Our characterization of the small DNA/RNA binding proteins can provide not only a new insight for molecular biology but also the comprehensive understanding of how we are able to design more potent, less toxic Toc-HDO drugs to achieve target gene expression regulation as one of the next-generation drugs.

## DATA AVAILABILITY

The datasets analyzed in the current study are available from the corresponding author upon reasonable request.

## Supplementary Material

gkab258_Supplemental_File
