# Supplementary material for: Short DNA/RNA heteroduplex oligonucleotide interacting proteins are key regulators of target gene silencing
Source: Nucleic Acids Res. 2021 Apr 30;49(9):4864–76. doi: 10.1093/nar/gkab258 (PMC8136785; doi:10.1093/nar/gkab258)
Supplement: gkab258_Supplemental_File [file gkab258_supplemental_file.pdf]

SUPPLEMENTARY DATA

Short DNA/RNA heteroduplex oligonucleotide interacting proteins are key regulators of target gene silencing

Ken Asada[1,2], Fumika Sakaue[1,2], Tetsuya Nagata[1,2], Ji-chun Zhang[1,2], Kie Yoshida-Tanaka[1,2], Aya Abe[1,2], Makiko Nawa[3], Kazutaka Nishina[1,2] and Takanori Yokota[1,2,*]

[1]Department of Neurology and Neurological Sciences, Graduate School of Medical and Dental Sciences, Tokyo Medical and Dental University, 1-5-45 Yushima, Bunkyo-ku, Tokyo 113-8519, Japan

[2]Center for Brain Integration Research, Tokyo Medical and Dental University, 1-5-45 Yushima, Bunkyo-ku, Tokyo 113-8519, Japan

[3]Laboratory of Cytometry and Proteome Research, Nanken-Kyoten and Research Core Center, Tokyo Medical and Dental University, 1-5-45 Yushima, Bunkyo-ku, Tokyo 113-8510, Japan

Table S1. Oligos used to generate recombinant proteins and shRNA vectors

**Primers to generate recombinant proteins**	**Primers used to generate recombinant proteins**
ANXA5 forward primer	5′ tttgctagcatggctacgagaggcact 3′
ANXA5 reverse primer	5′ tttgcggccgcgtcatcctcgcccccgca3′
APEX1 forward primer	5′ tttgctagcatgccaaagcggggaaag 3′
APEX1 reverse primer	5′ tttgcggccgccagtgctaggtaaagggt 3′
CA8 forward primer	5′ tttgctagcatggctgacctgagcttc 3′
CA8 reverse primer	5′ tttgcggccgcctgaaaggccgctcggat 3′
FEN1 forward primer	5′ tttggatccgctagcatgggaattcacggcctt 3′
FEN1 reverse primer	5′ tttgcggccgcttttccccttcggaactt 3′
**Primers to generate shRNA for stable cell lines**	**Primers used to generate shRNA for stable cell lines**
shCtrl forward primer	5′ gatcccaacaagatgaagagcaccaattcaagagattggtgctcttcatcttgttgtttttta 3′
shCtrl reverse primer	5′ agcttaaaaaacaacaagatgaagagcaccaatctcttgaattggtgctcttcatcttgttgg 3′
shLuciferase forward primer	5′ gatccgattatgtccggttatgtattcaagagatacataaccggacataatctttttta 3′
shLuciferase reverse primer	5′ agcttaaaaaagattatgtccggttatgtatctcttgaatacataaccggacataatcg 3′
shANXA5 forward primer	5′ gatccgcccttctgatgttctgtctattcaagagatagacagaacatcagaagggcttttttta 3′
shANXA5 reverse primer	5′ agcttaaaaaagcccttctgatgttctgtctatctcttgaatagacagaacatcagaagggcg 3′
shCA8 forward primer	5′ gatccagtgctatgttgaatccaattgtcccttcaagagagggacaattggattcaacatagcacttttttta 3′
shCA8 reverse primer	5′ agcttaaaaaaagtgctatgttgaatccaattgtccctctcttgaagggacaattggattcaacatagcactg 3′
shRNase H1 forward primer	5′ gatccgtagtgagcaaggacgcatttttcaagagaaaatgcgtccttgctcactactttttta 3′
shRNase H1 reverse primer	5′ agcttaaaaaagtagtgagcaaggacgcattttctcttgaaaaatgcgtccttgctcactacg 3′
shAPEX1 forward primer	5′ gatccgaaaggtttggattgggtaaattcaagagatttacccaatccaaacctttctttttta 3′
shAPEX1 reverse primer	5′ agcttaaaaaagaaaggtttggattgggtaaatctcttgaatttacccaatccaaacctttcg 3′
shFEN1 forward primer	5′ gatccgaatgacatcaagagctacttttcaagagaaagtagctcttgatgtcattctttttta 3′
shFEN1 reverse primer	5′ agcttaaaaaagaatgacatcaagagctactttctcttgaaaagtagctcttgatgtcattcg 3′

Table S2. Proteins identified by mass spectrometry

Gene name	Sequence coverage [%]	Score	No. of peptides
Anxa5	76.2	1591.85	35
Ca8	18.9	206.69	5
Gamt	13.6	108.96	2
Pebp1	22.5	83.23	3
Coq9	9.9	65.75	2
Hnmt	6.1	38.19	1
Blvrb	4.4	27.64	1

Table S3. Proteins identified from fractions by mass spectrometry

Fraction No.	Gene name	Sequence coverage [%]	Score	No. peptides
S1	*Anxa5*	38.2	665.11	16
S1	*Stard10*	41.9	560.89	15
S1	*Ywhang*	50.6	514.66	13
S1	*Ywhae*	43.9	415.84	13
S1	*Gstz1*	24.1	242.28	7
S1	*Phyh*	17.2	178.26	6
S1	*Ywhaz*	15.5	158.08	4
S1	*Nudt14*	16.2	108.17	3
S1	*Adprhl2*	12.2	101.87	5
S1	*Txn*	31.4	82.92	3
S1	*Rpl12*	9.1	72.48	1
S1	*Rbbp7*	2.4	70.37	1
S1	*Rpl13*	10	63.57	2
S1	*Otub1*	5.5	63.01	2
S1	*Rps18*	13.2	61.37	2
S1	*Rps5*	8.3	57.45	2
S1	*Ca8*	8.6	54.37	2
S1	*Sds*	7.3	46.29	2
S1	*Pcna*	8.0	38.2	2
S1	*Krt10*	3.7	36.71	2
S1	*Ran*	5.1	35.97	1
S1	*Cbr1*	3.2	35.89	1
S1	*Rpl31*	7.2	35.24	1
S1	*Nudt19*	2.0	33.99	1
S1	*Spr*	5.7	31.56	1
S1	*Rpl32-ps*	5.2	28.53	1
S1	*Krt2*	1.4	28.32	1
S1	*Kif26a*	0.6	28.20	1
S1	*Rps13*	4.6	28.03	1
S1	*Echdc3*	4.3	27.95	1
S1	*Rps3a*	9.1	26.50	1
S1	*Cnpy2*	6.6	23.83	1
S1	*Krt5*	2.1	23.72	1
S1	*Adck3*	1.6	22.66	1
S1	*Rbp1*	8.9	21.72	1
S1	*Son*	0.4	19.38	1
S1	*Ca3*	3.5	19.05	1
S1	*Eif5*	3.5	18.24	1
S1	*Rsp9*	4.1	18.1	1
S2	*Scp2*	8.4	115.13	6
S2	*Rps5*	14.7	110.51	3

S2	*Rps18*	19.1	104.93	3
S2	*Hebp1*	14.7	66.16	2
S2	*Eif6*	7.3	54.73	2
S2	*Anp32a*	7.7	53.03	2
S2	*Mup3*	6.0	43.31	1
S2	*Anp32b*	7.4	41.92	2
S2	*Apoe*	2.9	34.27	1
S2	*Gclm*	2.9	31.57	1
S2	*Rps13*	4.6	29.6	1
S2	*Rps19*	12.4	27.57	2
S3	*Hebp1*	27.4	94.24	4
S3	*Mup1*	10.0	51.98	2
S3	*Ptges3*	10.6	35.82	2
S3	*Rpl22*	19.5	35.79	2
S3	*Uchl3*	10.4	34.65	2

Table S4. Determined Bmax and Kd

ANXA5	ASO	HDO	Toc-HDO
One site -- Specific binding		Ambiguous	
**Best-fit values**			
Bmax	-11.7	~ -1.071e+015	2.42
Kd	-0.731	~ -2.271e+015	5.38
**95% CI (profile likelihood)**			
Bmax	-infinity to 45284117750	(Very wide)	-0.933 to 7.67
Kd	-infinity to 9789348076	(Very wide)	5.02 to 5.55
**Goodness of Fit**			
Degrees of Freedom	6	6	6
R squared	0.000706	0.0229	0.792
Sum of Squares	762	510	2850
Sy.x	11.3	9.22	21.8
**Number of points**			
# of X values	8	8	8
# Y values analyzed	8	8	8
**CA8**	**ASO**	**HDO**	**Toc-HDO**
One site -- Specific binding			
**Best-fit values**			
Bmax	-0.8752	2.72	0.7342
Kd	4.361	5.339	5.455
**95% CI (profile likelihood)**			
Bmax	-infinity to 0.07798	-0.868 to 8.98	-1.13 to 3.36
Kd	-infinity to N/A	4.76 to 5.56	5.27 to 5.57
**Goodness of Fit**			
Degrees of Freedom	6	6	6
R squared	0.1164	0.7147	0.8705
Sum of Squares	94.54	3071	1085
Sy.x	3.97	22.62	13.45
**Number of points**			
# of X values	8	8	8
# Y values analyzed	8	8	8
**APEX1**	**ASO**	**HDO**	**Toc-HDO**
One site -- Specific binding		Ambiguous	
Best-fit values			
Bmax	4.705	~ -0.0168	0.7023
Kd	5.298	~ 5.50	5.449
**95% CI (profile likelihood)**			
Bmax	1.08 to 9.79	(Very wide)	-0.470 to 2.19
Kd	5.01 to 5.45	(Very wide)	5.33 to 5.53
**Goodness of Fit**			
Degrees of Freedom	6	6	6
R squared	0.8632	0.9105	0.9336

Sum of Squares	2102	419.5	363.9
Sy.x	18.72	8.361	7.788
**Number of points**			
# of X values	8	8	8
# Y values analyzed	8	8	8
**FEN1**	**ASO**	**HDO**	**Toc-HDO**
One site -- Specific binding			
**Best-fit values**			
Bmax	5.78	1.067	2.411
Kd	5.267	5.428	5.336
**95% CI (profile likelihood)**			
Bmax	0.15 to 15.4	-2.27to 8.36	-1.08 to 9.28
Kd	4.61 to 5.40	4.21 to 5.69	4.42 to 5.58
**Goodness of Fit**			
Degrees of Freedom	6	6	6
R squared	0.729	0.5657	0.6412
Sum of Squares	5515	4947	3272
Sy.x	30.32	28.71	23.35
**Number of points**			
# of X values	8	8	8
# Y values analyzed	8	8	8

Table S5. Proteins identified from fractions by mass spectrometry

Fraction No.	Gene name	Sequence coverage [%]	Score	No. peptides
C1	*Ca8*	9.3	85.01	3
C1	*Gapdh*	9.0	42.6	3
C2	*TPM3*	9.1	53.39	3

A

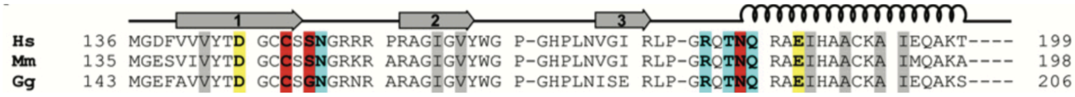

B

```
RNase H1: AF048993   VYWGPGHPLNVGIRLPGRQTNQRAE----IHAACKAIMQAKAQNISK
                     VY   G P  +       +++ +RAE    +   A +A M+ + +  +K
FEN1: NP_001258543   VYVFDGKPPQLKSGELAKRSERRAEAEKQLQQAQEAGMEEEVEKFTK
```

C

```
MGIHGLAKLI ADVAPSAIRE NDIKSYFGRK VAIDASMSIY QFLIAVRQGG
DVLQNEEGET TSHLMGMFYR TIRMMENGIK PVYVFDGKPP QLKSGELAKR
SERRAEAEKQ LQQAQEAGME EEVEKFTKRL VKVTKQHNDE CKHLLSLMGI
PYLDAPSEAE ASCAALAKAG KVYAAATEDM DCLTFGSPVL MRHLTASEAK
KLPIQEFHLS RVLQELGLNQ EQFVDLCILL GSDYCESIRG IGPKRAVDLI
QKHKSIEEIV RRLDPSKYPV PENWLHKEAQ QLFLEPEVLD PESVELKWSE
PNEEELVKFM CGEKQFSEER IRSGVKRLSK SRQGSTQGRL DDFFKVTGSL
SSAKRKEPEP KGPAKKKAKT GGAGKFRRGK
```

Figure S1. **Bioinformatics analysis of RNase H and FEN1.** (**A**) Sequence alignment of catalytic domains of multiple RNase H from (57). Conserved residues in the active site are highlighted in yellow, interaction with the RNA strand in red and interacting with the DNA in cyan. Conserved hydrophobic core residues are highlighted in gray. (**B**) Mouse FEN1 sequence (NP_001258543) was aligned to mouse RNase H1 (AF048993). (**C**) Known catalytic domain of FEN1 is highlighted in red (34). Active sites, interacting with DNA, and conserved hydrophobic core residues referring from RNase H1 sequence are highlighted in yellow, cyan, and gray.

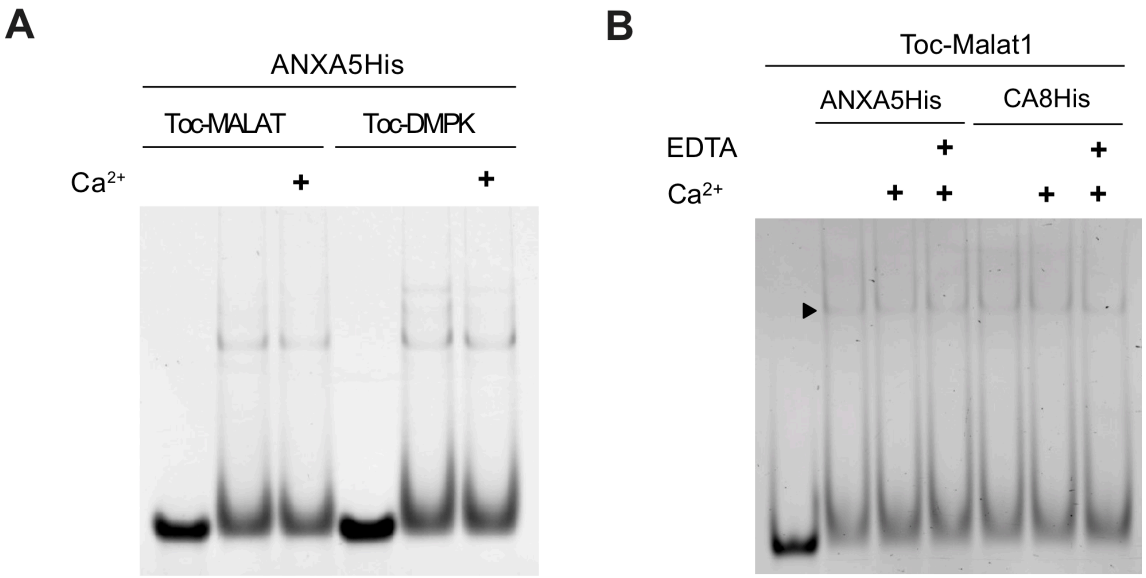

Figure S2. Calcium independent binding pattern of ANXA5 and CA8. (**A** and **B**) Toc-HDO binding assays with or without 5 mM Ca^{2+} with (A) ANXA5 (1,000 nM) or (B) ANXA5 and CA8 (1,000 nM each) in the presence of 2 mM Ca^{2+} and 2 mM EDTA.

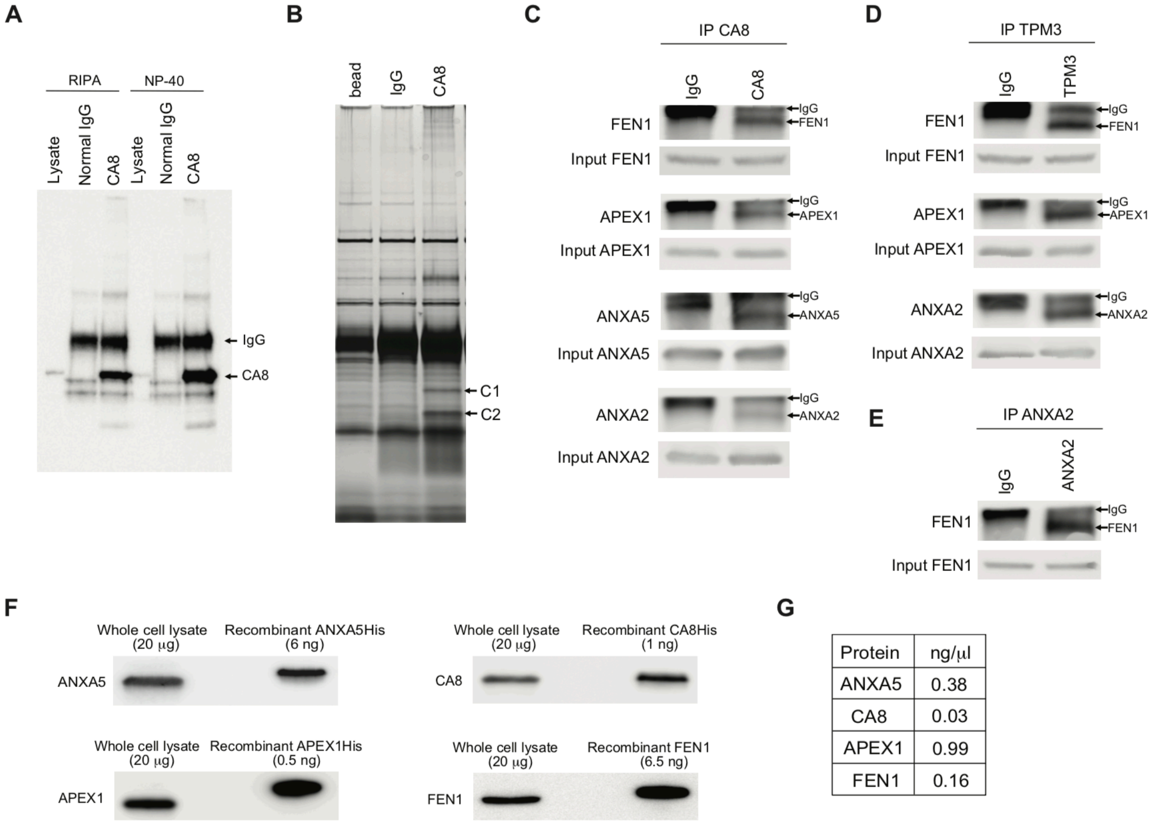

Protein	ng/μl
ANXA5	0.38
CA8	0.03
APEX1	0.99
FEN1	0.16

Figure S3. **The protein-protein interactions of the Toc-HDO binding proteins and the expression analysis of the Toc-HDO binding proteins.** (**A**) Mice liver samples were immunoprecipitated using an antibody against CA8 and immunoblotted with the CA8 antibody. Samples were lysed using either RIPA buffer or a nonionic detergent NP-40 buffer. (**B**) Immunoprecipitated samples were resolved by SDS-PAGE and visualized by silver staining. (**C**) Immunoprecipitation - western blotting analysis precipitated by (C) CA8, (D) TPM3, or (E) ANXA2. Each experiment was performed at least three times with similar results. (**F**) Analysis of Hepa 1-6 cellular protein expression levels resolved on SDS-PAGE with purified recombinant proteins. (**G**) Estimated expression levels of (F) analyzed by Image J software (NIH).
